# Supplementary material for: Histone deacetylase 1 maintains lineage integrity through histone acetylome refinement during early embryogenesis
Source: eLife. 2023 Mar 27;12:e79380. doi: 10.7554/eLife.79380 (PMC10079291; doi:10.7554/eLife.79380)
Supplement: Figure 1—source data 1. [file elife-79380-fig1-data1.zip › Cho_08-04-2022-RA-eLife-79380R1_Supporting_Zip_Document (2).pdf]

Unprocessed Western Blots:

<u>Western Blots in Figure 1A:</u>

Hdac1:

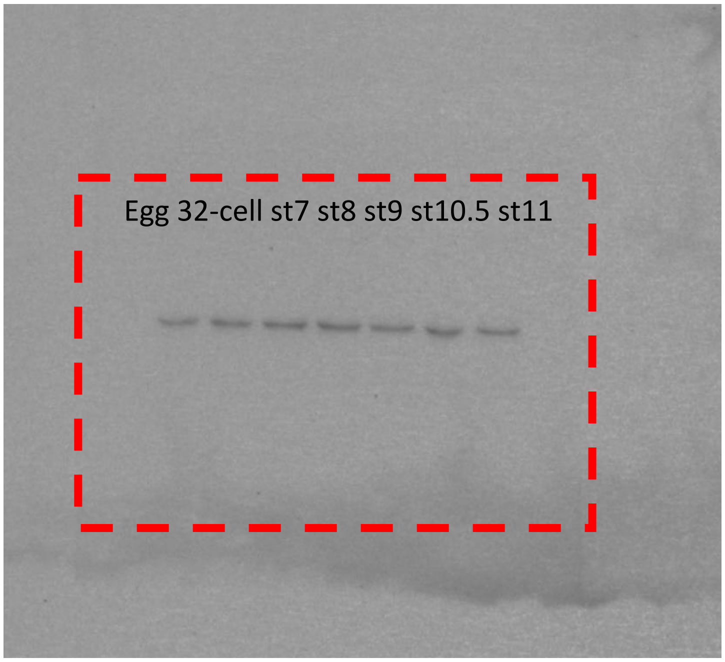

This is not developed on film, but by ChemiDoc MP Imaging system.

Hdac2 a-Tubulin

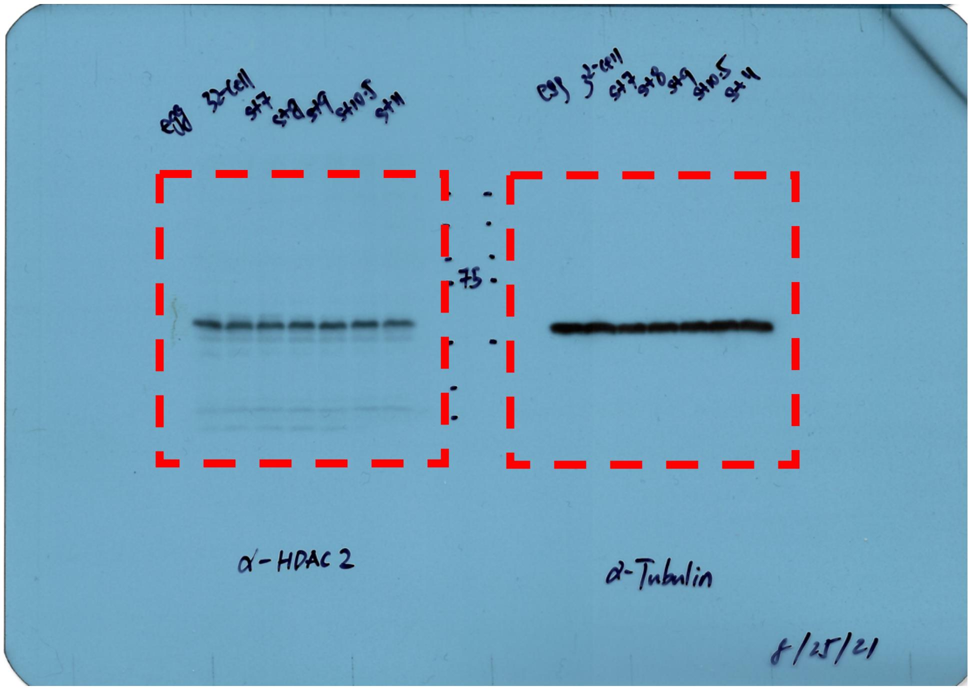

Figure 1. Hdac1 binds to genome gradually during early Xenopus development
(A) Western blot analyses showing Hdac1 and Hdac2 proteins over a time course of early development. a-tubulin is used as a loading control.
