# Supplementary material for: Histone deacetylase 1 maintains lineage integrity through histone acetylome refinement during early embryogenesis
Source: eLife. 2023 Mar 27;12:e79380. doi: 10.7554/eLife.79380 (PMC10079291; doi:10.7554/eLife.79380)
Supplement: Figure 4—source data 1. [file elife-79380-fig4-data1.zip › Cho_08-04-2022-RA-eLife-79380R1_Supporting_Zip_Document (8).pdf]

<u>Western Blots in Figure 4B:</u>

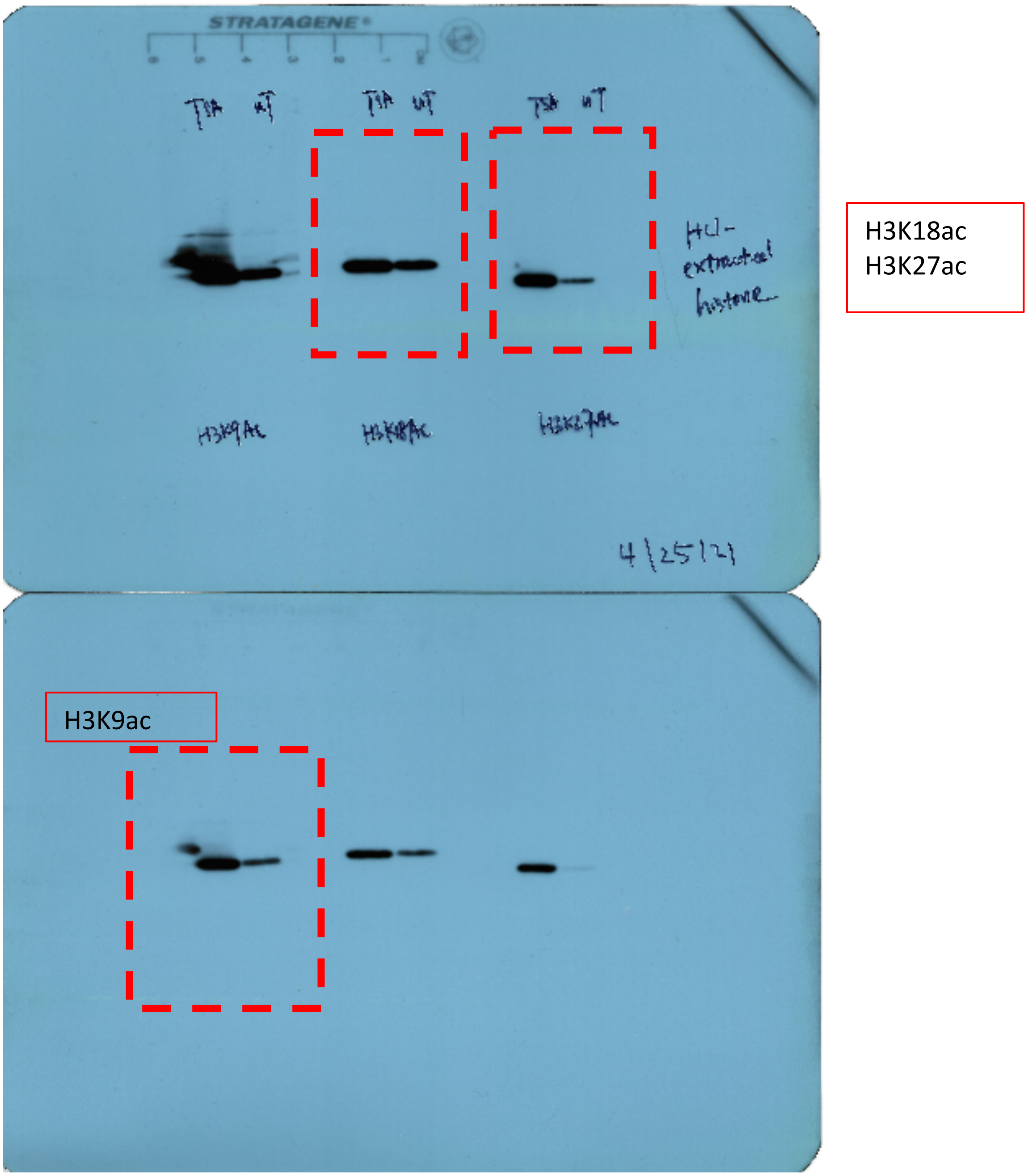

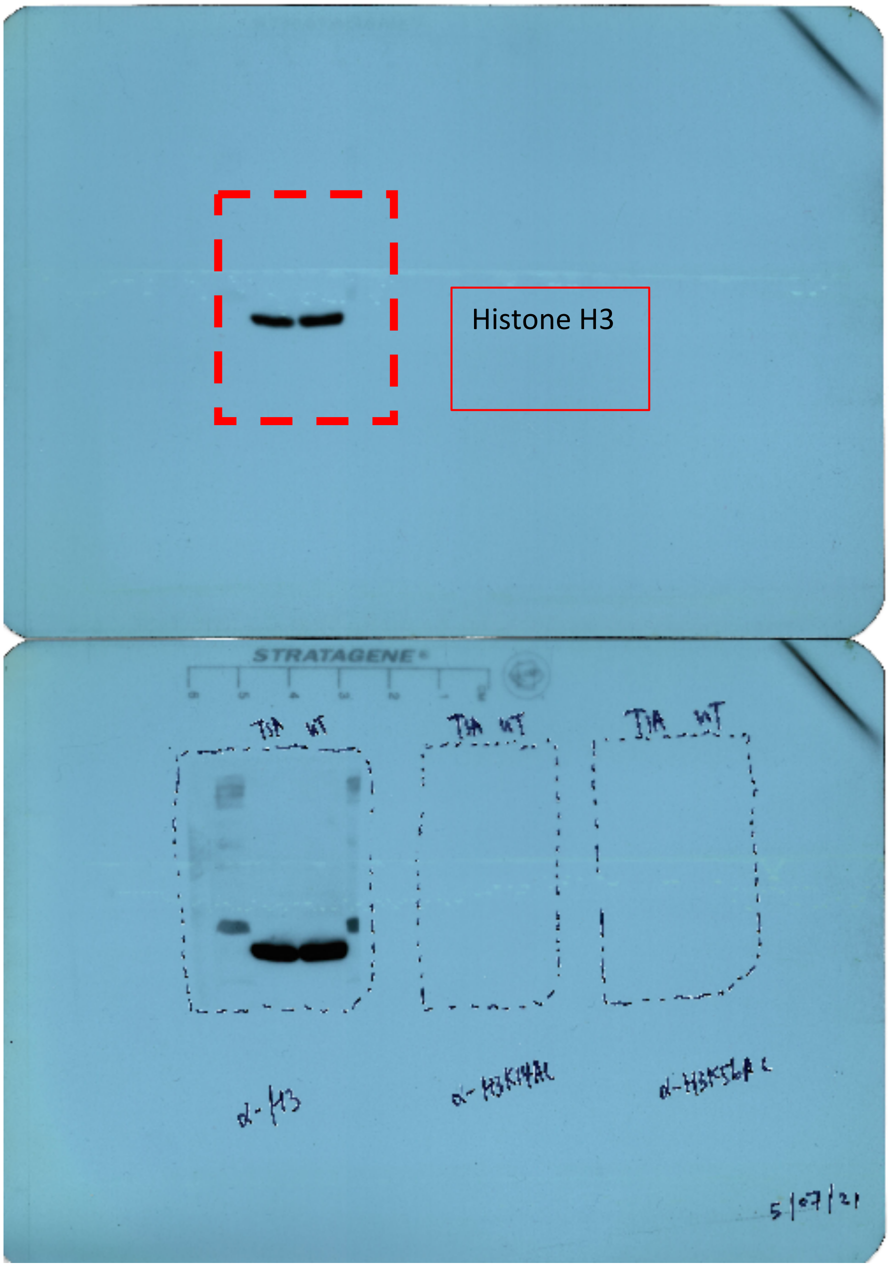

Figure 4: Hdac1 maintains differential H3 acetylomes etween germ layers. (B) Western blot analyses showing various histone acetylation modifications affected by HDAC inhibition. anti-H3 is used as a loading control.
