# Supplementary material for: Histone deacetylase 1 maintains lineage integrity through histone acetylome refinement during early embryogenesis
Source: eLife. 2023 Mar 27;12:e79380. doi: 10.7554/eLife.79380 (PMC10079291; doi:10.7554/eLife.79380)
Supplement: Figure 4—figure supplement 1—source data 1. [file elife-79380-fig4-figsupp1-data1.zip › Cho_08-04-2022-RA-eLife-79380R1_Supporting_Zip_Document (5).pdf]

Western Blots in Figure 4-figure supplement 1B

This is not developed on film, but by ChemiDoc MP Imaging system.

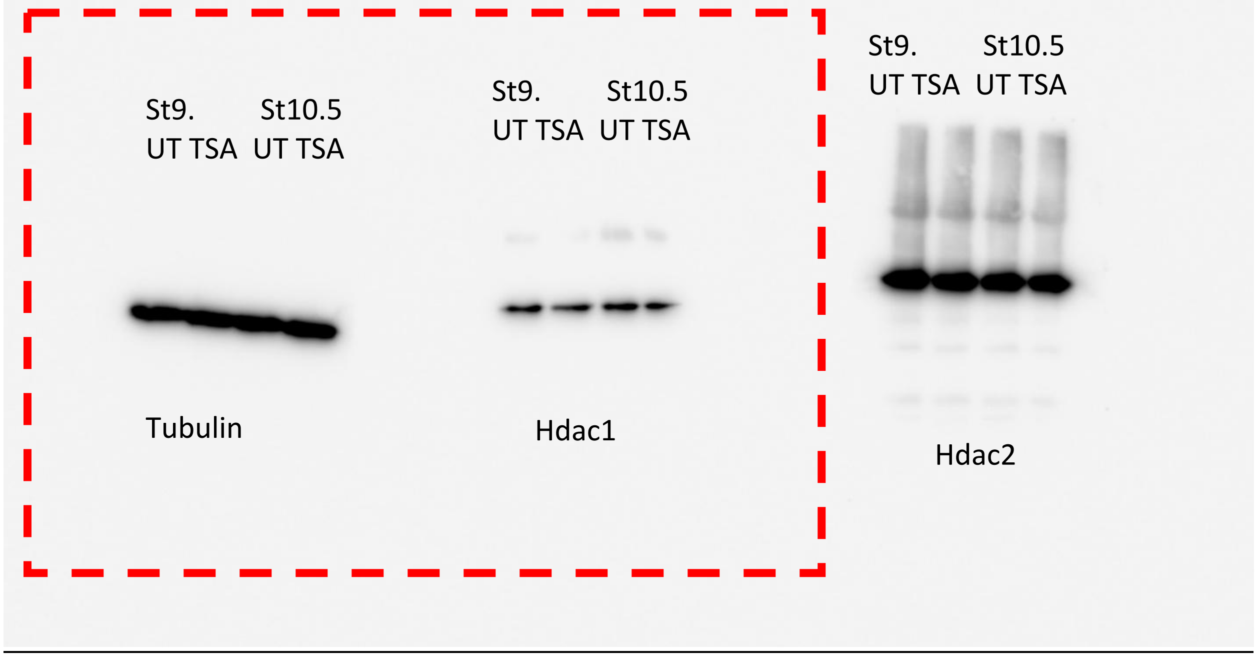

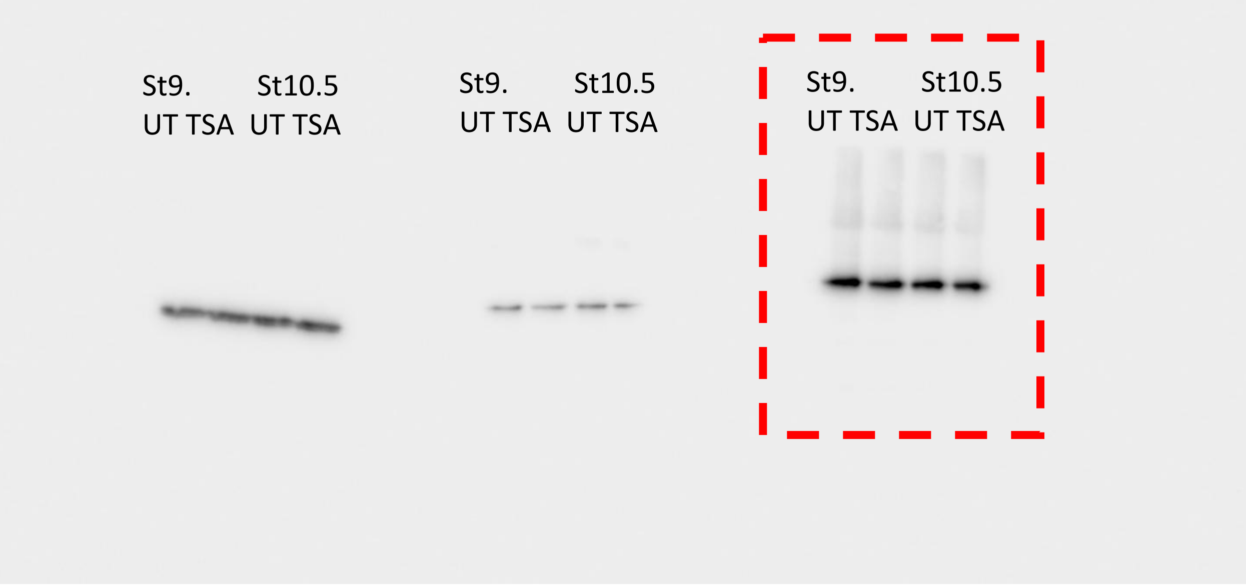

Figure 4–figure supplement 1. TSA mediated HDAC inhibition does not alter Hdac1 genomic occupancy. Western blot analyses showing protein levels of Hdac1 and Hdac2 upon TSA treatment in st9 and st10.5 embryos. b-Tubulin is used as a loading control.
