# Supplementary material for: Histone deacetylase 1 maintains lineage integrity through histone acetylome refinement during early embryogenesis
Source: eLife. 2023 Mar 27;12:e79380. doi: 10.7554/eLife.79380 (PMC10079291; doi:10.7554/eLife.79380)
Supplement: Supplementary file 1. [file elife-79380-supp1.docx]

**Supplementary File 1**

Top motifs detected in HDAC1 peaks at stage 8, 9 and 10 embryos.

**Top 50 motifs detected in st8 Hdac1 peaks (500 bps) ranked by FDR**

| **motif_id** | **motif_alt_id** | **consensus** | **FDR** |
| --- | --- | --- | --- |
| MA0479.1 | FOXH1 | BNSAATCCACA | 5.50E-42 |
| MA0507.2 | POU2F2 | AWTATGCAAATKAG | 1.50E-17 |
| MA0784.2 | POU1F1 | ANCTMATTWGCATAWT | 1.60E-16 |
| UP00191_1 | Pou2f2_3748.1 | BHDTATGCAAATKARV | 1.10E-14 |
| MA1962.1 | POU2F1::SOX2 | MATTTRCATMACAATRG | 5.00E-14 |
| UP00179_1 | Pou2f3_3986.2 | THKTATGCAAATKMDM | 1.20E-13 |
| MA0627.2 | POU2F3 | NWTATGCAAATNH | 9.90E-13 |
| POU1F1_DBD_2 | | AWTATGCWAATKAG | 1.70E-12 |
| MA0789.1 | POU3F4 | TATGCWAAT | 1.70E-11 |
| POU3F4_DBD_1 | | TATGCWAAT | 1.10E-10 |
| MA0786.1 | POU3F1 | WTATGCWAATKW | 3.00E-10 |
| Pou2f2_DBD_2 | | TATGCAAAT | 6.40E-10 |
| POU3F1_DBD_1 | | WTATGCWAATKW | 7.20E-10 |
| POU3F2_DBD_2 | | WTATGCWAATKA | 1.30E-09 |
| MA0613.1 | FOXG1 | RTAAACAW | 1.50E-09 |
| POU2F2_DBD_1 | | DTATGCWAATK | 1.70E-09 |
| POU2F1_DBD_1 | | AWTATGCWAATK | 2.60E-09 |
| MA0142.1 | Pou5f1::Sox2 | CWTTGTYATGCAAAT | 8.70E-09 |
| MA0785.1 | POU2F1 | AWTATGCWAATK | 9.20E-09 |
| POU5F1P1_DBD_1 | | TATGCWAAT | 9.90E-09 |
| MA0792.1 | POU5F1B | TATGCWAAT | 1.20E-08 |
| MA0787.1 | POU3F2 | WTATGCWAATKA | 1.30E-08 |
| POU2F3_DBD_1 | | TATGCWAAT | 4.00E-08 |
| MA1115.1 | POU5F1 | WHATGCAAATN | 5.10E-08 |
| MA0788.1 | POU3F3 | WWTATGCWAATTW | 1.30E-07 |
| POU3F3_DBD_1 | | WWTATGCWAATTW | 3.20E-07 |
| UP00049_1 | Sp100_primary | RWTWHNCGGAAAWT | 1.10E-06 |
| PAX6_DBD |  | TTTCACGCWTGAVTGMRYW | 3.00E-06 |
| MA0527.1 | ZBTB33 | BTCTCGCGAGABYTS | 3.20E-06 |
| MA0849.1 | FOXO6 | GTAAACA | 4.80E-06 |
| FOXI1_full_1 |  | GTAAACA | 5.50E-06 |
| FOXO6_DBD_2 | | GTAAACA | 5.70E-06 |
| MA1103.2 | FOXK2 | DDGTAAACANN | 1.00E-05 |
| MA0042.2 | FOXI1 | GTAAACA | 1.40E-05 |
| MA0670.1 | NFIA | NDTGCCAAND | 3.20E-05 |
| NFIA_full_2 |  | NDTGCCAAND | 3.20E-05 |
| UP00259_1 | Hoxb6_3428.2 | WWTKNGTAATTACNDN | 3.50E-05 |
| PROX1_DBD |  | YAAGACGYCTTA | 4.50E-05 |
| MA0651.2 | HOXC11 | AGGTCGTAAAAH | 5.50E-05 |
| UP00211_1 | Pou3f3_3235.2 | WANDTATGCATAATDDA | 7.50E-05 |
| MA0873.1 | HOXD12 | RGTCGTAAAAH | 7.70E-05 |
| NFIX_full_4 |  | YTGGCAHNDTGCCAA | 1.90E-04 |
| MA0705.1 | Lhx8 | CTAATTAV | 1.90E-04 |
| MA1643.1 | NFIB | NNCCTGGCAYNGTGCCAAGNN | 2.20E-04 |
| Lhx8_DBD_2 |  | CTAATTAV | 2.30E-04 |
| MA1527.1 | NFIC | NTTGGCDNNRTGCCARN | 2.70E-04 |
| FOXO4_DBD_2 | | GTAAACA | 2.70E-04 |
| MA0852.2 | FOXK1 | NRDGTAAACAAGNN | 2.90E-04 |
| HOXA2_DBD |  | NSTMATTAVS | 3.40E-04 |
| NFIX_full_1 |  | YTGGCDNNNTGCCAA | 3.60E-04 |

**Top 50 motifs detected in st9 Hdac1 peaks (500 bps) ranked by FDR**

| **motif_id** | **motif_alt_id** | **consensus** | **FDR** |
| --- | --- | --- | --- |
| MA0142.1 | Pou5f1::Sox2 | CWTTGTYATGCAAAT | 5.90E-121 |
| MA1962.1 | POU2F1::SOX2 | MATTTRCATMACAATRG | 4.10E-111 |
| MA0515.1 | Sox6 | CCWTTGTYYY | 2.70E-97 |
| MA0867.2 | SOX4 | RAACAAAGRV | 1.80E-92 |
| POU1F1_DBD_2 | | AWTATGCWAATKAG | 1.50E-86 |
| MA1152.1 | SOX15 | CYWTTGTTHW | 1.30E-84 |
| UP00062_1 | Sox4_primary | HNNWRAACAAAGRRNHW | 1.50E-84 |
| UP00179_1 | Pou2f3_3986.2 | THKTATGCAAATKMDM | 1.60E-81 |
| UP00191_1 | Pou2f2_3748.1 | BHDTATGCAAATKARV | 9.10E-80 |
| SOX9_DBD |  | DAACAATRG | 9.80E-79 |
| MA0787.1 | POU3F2 | WTATGCWAATKA | 2.50E-78 |
| POU3F2_DBD_2 | | WTATGCWAATKA | 1.10E-77 |
| UP00030_1 | Sox11_primary | HNDARAACAAAGRANNH | 2.30E-77 |
| MA0479.1 | FOXH1 | BNSAATCCACA | 8.00E-77 |
| MA0868.2 | SOX8 | MGAACAATRG | 1.50E-76 |
| MA0784.2 | POU1F1 | ANCTMATTWGCATAWT | 1.10E-75 |
| FOXJ2_DBD_2 | | RTAAACAA | 1.50E-75 |
| MA0087.2 | Sox5 | VRVAACAATGGNN | 2.30E-75 |
| MA0789.1 | POU3F4 | TATGCWAAT | 1.40E-74 |
| MA0507.2 | POU2F2 | AWTATGCAAATKAG | 3.80E-73 |
| FOXO1_DBD_1 | | GTAAACAW | 2.70E-72 |
| POU3F4_DBD_1 | | TATGCWAAT | 3.30E-72 |
| MA0613.1 | FOXG1 | RTAAACAW | 3.80E-69 |
| MA0785.1 | POU2F1 | AWTATGCWAATK | 7.40E-69 |
| MA0514.2 | Sox3 | DNACAATGGNN | 1.60E-67 |
| MA0614.1 | Foxj2 | RTAAACAA | 2.50E-67 |
| MA1120.1 | SOX13 | DVACAATGGNN | 4.90E-67 |
| POU2F2_DBD_1 | | DTATGCWAATK | 1.10E-66 |
| POU2F1_DBD_1 | | AWTATGCWAATK | 1.20E-66 |
| UP00016_2 | Sry_secondary | HNDNNRAACAATDRVHG | 2.10E-66 |
| UP00004_2 | Sox14_secondary | NKBASACAATRGNBN | 6.30E-66 |
| MA0078.2 | Sox17 | NNAGAACAATGGNN | 2.90E-65 |
| Foxj3_DBD_3 |  | RTAAACAA | 3.20E-65 |
| MA0852.2 | FOXK1 | NRDGTAAACAAGNN | 3.50E-65 |
| MA0786.1 | POU3F1 | WTATGCWAATKW | 1.60E-64 |
| POU3F1_DBD_1 | | WTATGCWAATKW | 1.90E-64 |
| UP00041_1 | Foxj1_primary | ANHRTAAACAAANNND | 1.80E-63 |
| FOXO6_DBD_2 | | GTAAACA | 3.30E-62 |
| MA0849.1 | FOXO6 | GTAAACA | 1.10E-61 |
| POU2F3_DBD_1 | | TATGCWAAT | 1.80E-60 |
| MA1115.1 | POU5F1 | WHATGCAAATN | 1.90E-60 |
| Foxg1_DBD_3 | | GTAAACA | 3.20E-59 |
| MA0143.4 | SOX2 | DNACAATGGNN | 5.00E-58 |
| Pou2f2_DBD_2 | | TATGCAAAT | 1.50E-57 |
| MA0851.1 | Foxj3 | NDAADGTAAACAAANNM | 2.50E-57 |
| MA0792.1 | POU5F1B | TATGCWAAT | 6.50E-57 |
| POU5F1P1_DBD_1 | | TATGCWAAT | 7.30E-57 |
| MA0627.2 | POU2F3 | NWTATGCAAATNH | 1.70E-56 |
| MA0033.2 | FOXL1 | RTAAACA | 1.90E-56 |
| MA1563.2 | SOX18 | AACAATDV | 2.10E-56 |

**Top 50 motifs detected in st10.5 Hdac1 peaks (500 bps) ranked by FDR**

| **motif_id** | **motif_alt_id** | **consensus** | **FDR** |
| --- | --- | --- | --- |
| ZIC4_DBD |  | GRCCCCCCGCKGYGH | 7.10E-199 |
| MA0751.1 | ZIC4 | GRCCCCCCGCKGYGH | 8.00E-197 |
| ZIC3_full |  | GACCCCCCGCTGYGH | 3.70E-172 |
| Zic3_DBD |  | GACCCCCYGCTGYGH | 2.80E-165 |
| SP1_DBD |  | RCCMCRCCCMC | 3.60E-160 |
| SP3_DBD |  | VCCACGCCCMC | 3.40E-157 |
| MA1629.1 | Zic2 | NDCACAGCAGGDRG | 4.20E-155 |
| MA1653.1 | ZNF148 | YBCCCCTCCCCC | 5.30E-153 |
| MA0740.2 | KLF14 | KGGGCGGGG | 5.40E-153 |
| MA0599.1 | KLF5 | GCCCCDCCCH | 1.40E-151 |
| MA1961.1 | PATZ1 | SSGGGGMGGGGS | 2.20E-149 |
| MA1564.1 | SP9 | RCCACGCCCMCY | 1.80E-148 |
| UP00002_1 | Sp4_primary | BDHMMCGCCCCCTYNNB | 7.00E-148 |
| MA0746.2 | SP3 | NGCCACGCCCMCH | 3.10E-147 |
| MA1511.2 | KLF10 | GGGGCGGGG | 5.00E-146 |
| MA0697.2 | Zic3 | CNCAGCAGGAGNN | 2.00E-145 |
| MA0516.3 | SP2 | GGGGCGGGG | 4.20E-145 |
| MA1965.1 | SP5 | CBCCTCCCHS | 6.80E-145 |
| MA1512.1 | KLF11 | SCCACGCCCMC | 1.20E-143 |
| MA1515.1 | KLF2 | NRCCACRCCCH | 2.10E-141 |
| MA0696.1 | ZIC1 | GACCCCCYGCTGTG | 1.60E-140 |
| ZIC1_full |  | GACCCCCYGCTGTG | 1.70E-139 |
| MA0079.5 | SP1 | GGGGCGGGG | 9.30E-138 |
| MA0471.2 | E2F6 | VVDGGCGGGAAVV | 1.30E-137 |
| MA0039.4 | KLF4 | NNCCCCACCCHN | 1.80E-137 |
| MA1713.1 | ZNF610 | SSCGCCGCTCCSSS | 2.20E-132 |
| UP00093_1 | Klf7_primary | TNRRCCMCGCCCHYHD | 1.20E-130 |
| MA1959.1 | KLF7 | DGGGCGGGG | 8.20E-130 |
| KLF16_DBD |  | GMCACGCCCCC | 1.50E-129 |
| MA0162.4 | EGR1 | VCMCGCCCACGCVS | 1.20E-128 |
| MA0528.2 | ZNF263 | VNGGGGAGGASG | 1.50E-128 |
| MA0742.2 | KLF12 | GGGGCGGGG | 2.70E-128 |
| MA0741.1 | KLF16 | GMCACGCCCCC | 1.50E-125 |
| MA0515.1 | Sox6 | CCWTTGTYYY | 1.30E-124 |
| UP00057_2 | Zic2_secondary | NHDCVCAGCAKGNRW | 3.80E-124 |
| UP00102_1 | Zic1_primary | BHCCCCCGGGGGGG | 4.10E-124 |
| MA1522.1 | MAZ | BBCCCCTCCCY | 1.30E-123 |
| UP00007_1 | Egr1_primary | NCCGCCCCCGCAYK | 1.10E-121 |
| UP00057_1 | Zic2_primary | NMCCCCCGGGGGGGT | 2.80E-120 |
| UP00006_1 | Zic3_primary | BMCCCCCGGGGGGGB | 2.90E-120 |
| UP00102_2 | Zic1_secondary | YHDCRCAGCAGGMVD | 9.80E-120 |
| MA1102.2 | CTCFL | NSCAGGGGGCGS | 1.90E-116 |
| TFAP2A_DBD_2 | | HGCCYSAGGCD | 1.80E-115 |
| MA1122.1 | TFDP1 | VSGCGGGAAVN | 2.30E-115 |
| UP00005_1 | Tcfap2a_primary | WDTSCCYSRGGSRAN | 3.60E-114 |
| MA0814.2 | TFAP2C | NNGCCTGAGGCGNN | 3.90E-114 |
| Tcfap2a_DBD_2 | | HGCCYSAGGCD | 9.80E-113 |
| UP00000_2 | Smad3_secondary | KHHNCCCCGCCAMYYYB | 2.30E-112 |
| TFAP2A_DBD_1 | | YGCCCBVRGGCR | 3.90E-112 |
| MA1628.1 | Zic1::Zic2 | CVCAGCAGGNV | 8.70E-112 |
