# Supplementary material for: Histone deacetylase 1 maintains lineage integrity through histone acetylome refinement during early embryogenesis
Source: eLife. 2023 Mar 27;12:e79380. doi: 10.7554/eLife.79380 (PMC10079291; doi:10.7554/eLife.79380)
Supplement: Supplementary file 2. [file elife-79380-supp2.docx]

**Supplementary File 2**

References for known germ-layer functioning genes in Figure 5-figure supplement 1E.

| Gene type | Gene symbol | Gene function (Germ layer specification) | PubMed reference |
| --- | --- | --- | --- |
| Up in AC | *adrb2* | Axis posteriorization via mesoderm | PMID: 23452088 |
| Up in AC | *foxc1* | Modulate meso/endodermal genes | PMID: 17705306 |
| Up in AC | *hhex* | Organizer suppression and anterior specification | PMID: 10804173 |
| Up in AC | *osr1* | Limit endoderm differentiation from mesendoderm | PMID: 18787069 |
| Up in AC | *snai1* | Embryonic mesoderm and neural crest formation | PMID: 21715424 |
| Up in AC | *vegfa* | Pattern dorsal aorta/ vasculature | PMID: 9729498 |
| Down in AC | *bambi* | TGFb inhibitor, blocks mesoderm in ectoderm | PMID: 10519551 |
| Down in AC | *dlx5* | Define the lateral boundary between the neural plate and epidermis | PMID: 11599044 |
| Down in AC | *foxi4.2* | Ventral ectoderm specification | PMID: 16079156 |
| Down in AC | *hes4* | Neural crest formation | PMID: 17436284 |
| Down in AC | *irx2* | Control the differentiation of neural plate | PMID: 9427752 |
| Down in AC | *tfap2a* | Neural crest development in ectoderm | PMID: 17724731 |
| Up in VG | *cdx2* | Organizer-specific, posterior axis | PMID: 1677215 |
| Up in VG | *esr5* | Mesoderm area restriction | PMID: 21425079 |
| Up in VG | *jun* | Regulate neurogenesis by inducing FoxD5b | PMID: 24623078 |
| Up in VG | *numbl* | Primary neurogenesis | PMID: 24125469 |
| Down in VG | *foxa2* | Limit extend of mesoderm in endoderm | PMID: 14697355 |
| Down in VG | *gata5* | Maintenance of endodermal gene expression | PMID: 15659482 |
| Down in VG | *pnhd* | Activate a subset of mesodermal genes | PMID: 32859582 |
| Down in VG | *osr1* | Limit endoderm differentiation from mesendoderm | PMID: 18787069 |
