# Supplementary material for: Histone deacetylase 1 maintains lineage integrity through histone acetylome refinement during early embryogenesis
Source: eLife. 2023 Mar 27;12:e79380. doi: 10.7554/eLife.79380 (PMC10079291; doi:10.7554/eLife.79380)
Supplement: Supplementary file 3. [file elife-79380-supp3.docx]

**Supplementary File 3**

ChIP-qPCR Primer Sequences

| Primer targets | Forward Sequence | Reverse Sequence |
| --- | --- | --- |
| *alkbh2* intron | GTCAGCATGGTGGATGTAGT | GTCTGATCAGTGTCTCCGATT |
| *bmp4* intron | TGCTAATAAGCGTGCCTTTG | AAGGAGGACCAGCCTATTCA |
| *erfl* intron | ATTAGCACTGGGACGGCTCT | GCTGTCCCAGTGCTGATTATAG |
| *fgf5* 5’-up | TTAGCATGCAGAGAAGAGGC | TATTACATTCTCGCACGGCT |
| *foxa4* intron | ATCAGCACTTTTGTGTCCTT | ACTGTTTGGGGTTGGAATG |
| *foxi4.2* pro | AGGGTAACGGTGCGCACACA | GCAGTGCCAGACAGGGGAAG |
| *gdnf* 5’-up | GCACAGGATATGGGCCATTG | CAGGGCAGCCACTGGTGTAT |
| *hhex* promoter | CCGTACGGAGAGGTGACATG | TGACAGAGCAGGAAGTGGCT |
| *hoxb3* intron | GTAGATCTGTCAGGGAGAAA | GCCATTGATTGAGATCCGTA |
| *hspa4 3’UTR* | AGCATTGAAGATGGTCATTCG | TTGATAGGTACCGTTCGCCAT |
| *insm2* exon | AGTGTCCCGTTGAGTTTGCT | CTGGGAGATGGTGCTGATCG |
| *klf11* exon | TCCCATTTCTGTTCCTGTGT | GGAGAATAGGCTTTACTGTATTA |
| *miR428a* | TCTAGTTCGGTTGCTGAGTG | CTCTGGTTTGGCCAATCAGG |
| *not* 5’-up | TCAGGGTGCCTCTCCCATAC | CAAGGCAGATGTTAATGGCCC |
| *set* 3’-down | TGCAGCCCTGTTGTATAAAG | CAGATTATGATTGGCTGCCG |
| *snai1* intron | CGCACCATTAGCAATTCATGA | GGAAGGGCAATCTAAACAAG |
| *sox17b.2* 5’-up | AGCACCTAGGATAGTGTCAG | TTGAGAGGCTGTTATAGGCA |
| *sp8* 3’-down | ATGTGCTAAGTGGGGCTCAT | GGACACTCAGTGGTCACTGT |
| *tbxt.2* intron | ATTGGATTGGAACACTGGGA | AAGGTCTATTGACTCTGTTTCTC |
