# Supplementary material for: Histone deacetylase 1 maintains lineage integrity through histone acetylome refinement during early embryogenesis
Source: eLife. 2023 Mar 27;12:e79380. doi: 10.7554/eLife.79380 (PMC10079291; doi:10.7554/eLife.79380)
Supplement: Supplementary file 4. [file elife-79380-supp4.docx]

**Supplementary File 4**

| Sample | Reads to frog | Ratio | Reads to H2A.V peaks | Ratio | Merged Reps Ratio |
| --- | --- | --- | --- | --- | --- |
| AC_DMSO_1 | 43417738 | *1.17* | 647615 | *4.20* | *3.86* |
| AC_DMSO_2 | 47280904 | *1.27* | 581133 | *3.54* |  |
| AC_TSA_1 | 127763576 | *3.43* | 242780 | *1.48* | *1.34* |
| AC_TSA_2 | 86344294 | *2.32* | 186614 | *1.14* |  |
| VG_DMSO_1 | 41215108 | *1.11* | 691340 | *4.21* | *3.95* |
| VG_DMSO_2 | 37237950 | *1.00* | 601379 | *3.66* |  |
| VG_TSA_1 | 124387262 | *3.34* | 202869 | *1.23* | *1.13* |
| VG_TSA_2 | 101907192 | *2.74* | 164375 | *1.00* |  |

: Normalization Factors of ChIP spike-in strategy. Table listing all spike-in normalization factors. Reads mapped to *Xenopus tropicalis* (frog) for all second replicate samples are already **downsampled to 25%**. The lowest number of reads in each mapping is set to the baseline of 1. The ratios for reads mapped to H2Av peaks are used for all later calculations. The ratios for reads between replicate-merged samples are calculated as: $Merged bam rep ratio= Ratio\left( H2A.V_{rep1} \right)\times\frac{\mathrm{Ratio}\left( \mathrm{frog}_{rep1} \right)}{\mathrm{Ratio}\left( \mathrm{frog}_{rep1} \right)+Ratio\left( \mathrm{frog}_{rep2} \right)}\mathbf{+}\mathrm{Ratio}\left( H2A.V_{rep2} \right)\times\frac{\mathrm{Ratio}\left( \mathrm{frog}_{rep2} \right)}{\mathrm{Ratio}\left( \mathrm{frog}_{rep1} \right)+Ratio\left( \mathrm{frog}_{rep2} \right)}$ .
