# Supplementary material for: Histone deacetylase 1 maintains lineage integrity through histone acetylome refinement during early embryogenesis
Source: eLife. 2023 Mar 27;12:e79380. doi: 10.7554/eLife.79380 (PMC10079291; doi:10.7554/eLife.79380)
Supplement: Supplementary file 5. [file elife-79380-supp5.docx]

**Supplementary File 5**

p-Values from Fisher’s exact test for proportions of TSA/VPA co-regulated genes in different spatial categories.

Class 1 Genes: Figure 5F

| Gene *type* | Num. of genes in *type* | Num. of ref (All) genes | *p*-value |
| --- | --- | --- | --- |
| Up in AC: AC enriched | 9 | 3036 | 0.64 |
| Up in AC: MZ enriched | 7 | 965 | 0.035 |
| Up in AC: VG enriched | 43 | 6170 | **6.1e-8** |
| Down in AC: AC enriched | 34 | 3036 | **<2.2e-16** |
| Down in AC: MZ enriched | 14 | 965 | **1.9e-8** |
| Down in AC: VG enriched | 12 | 6170 | 0.72 |
| Up in VG: AC enriched | 8 | 3036 | 5.3e-3 |
| Up in VG: MZ enriched | 3 | 965 | 0.06 |
| Up in VG: VG enriched | 4 | 6170 | 0.86 |
| Down in VG: AC enriched | 10 | 3036 | 0.066 |
| Down in VG: MZ enriched | 10 | 965 | **1.6e-5** |
| Down in VG: VG enriched | 31 | 6170 | **3.1e-8** |

Class 3 Genes: Figure 5: figure supplementary 5F

| Gene *type* | Num. of genes in *type* | Num. of ref (All) genes | *p*-value |
| --- | --- | --- | --- |
| Up in AC: AC enriched | 17 | 3036 | 2.4e-3 |
| Up in AC: MZ enriched | 10 | 965 | **2.6e-4** |
| Up in AC: VG enriched | 35 | 6170 | **2.2e-6** |
| Down in AC: AC enriched | 51 | 3036 | **<2.2e-16** |
| Down in AC: MZ enriched | 7 | 965 | 9.8e-3 |
| Down in AC: VG enriched | 5 | 6170 | 1.0 |
| Up in VG: AC enriched | 2 | 3036 | 0.37 |
| Up in VG: MZ enriched | 3 | 965 | 6.6e-3 |
| Up in VG: VG enriched | 3 | 6170 | 0.49 |
| Down in VG: AC enriched | 16 | 3036 | **2.1e-7** |
| Down in VG: MZ enriched | 8 | 965 | **2.1e-5** |
| Down in VG: VG enriched | 9 | 6170 | 0.36 |

Class 1 Genes: Figure 5F: Gene type explanation: Up in AC: AC enriched = The expression of genes is normally enriched in ectodermal cells, and these genes are found to be upregulated in animal cap tissues upon HDACi (TSA and VPA)

Class 3 Genes: Figure 5-figure supplement 5F. Gene type explanation: Up in AC: AC enriched = The expression of genes is normally enriched in ectodermal cells, and these genes are found to be upregulated in animal cap tissues upon HDACi (TSA and VPA)
